# Data on the *Lignosus rhinocerotis* water soluble sclerotial extract affecting intracellular calcium level in rat dorsal root ganglion cells

**DOI:** 10.1016/j.dib.2018.04.033

**Published:** 2018-04-14

**Authors:** Mei-Kee Lee, Paul Millns, Yvonne Mbaki, Szu-Ting Ng, Chon-Seng Tan, Kuan-Hon Lim, Sue-Mian Then, Suresh Kumar Mohankumar, Kang-Nee Ting

**Affiliations:** a Faculty of Science, University of Nottingham Malaysia Campus, 43500 Semenyih, Malaysia; b School of Life Sciences, University of Nottingham, Queen's Medical Centre, Nottingham, NG7 2UH, UK; c LiGNO Biotech Sdn Bhd, 43300 Balakong Jaya, Malaysia; d JSS College of Pharmacy, Rocklands, Ootacamund 643001, Tamil Nadu, India; e College of Jagadguru Sri Shivarathreeshwara University, Mysuru, Karnataka, India

**Keywords:** *Lignosus rhinocerotis*, Medicinal mushroom, Bronchodilators, Calcium dynamics

## Abstract

The data in this article contain supporting evidence for the research manuscript entitled “Bronchodilator effects of *Lignosus rhinocerotis* extract on rat isolated airways is linked to the blockage of calcium entry” by Lee et al. (2018) [1]. The data were obtained by calcium imaging technique with fluorescent calcium indicator dyes, Fura 2-AM, to visualize calcium ion movement in the rat dorsal ganglion (DRG) cells. The effects of *L. rhinocerotis* cold water extract (CWE^1^) on intracellular calcium levels in the DRG cells were presented.

**Specifications table**

**Table:** 

Subject area	*Pharmacology*
More specific subject area	*Ethnopharmacology*
Type of data	*Trace recording, graph*
How data was acquired	Calcium imaging assay
	Leica DM-IRB and DM-IL microscope; QImaging Retiga-EXi camera. (Leica microsystems, Germany)
Data format	*Raw and analyzed*
Experimental factors	*DRG neuronal cells isolated from Sprague Dawley rats were pre-loaded with Fura 2-AM solution before exposure to potassium chloride (KCl)* (30 mM) *to evoke intracellular calcium increase.*
Experimental features	*Cells were infused with CWE* (3.5 mg/ml) *or nifedipine* (30 μM) *for* 30 min *before the second exposure to KCl.*
Data source location	*Sample of Lignosus rhinocerotis obtained from Ligno Biotech, Malaysia.*
	*Experiments were carried out at the University of Nottingham, UK.*
Data accessibility	*The data is presented in this article*
Related research article	*Bronchodilator effects of Lignosus rhinocerotis extract on rat isolated airways is linked to the blockage of calcium entry*[1]

**Value of the data**

• The data presented here describes the effect of *L. rhinocerotis* on intracellular calcium increase induced by high potassium treatment into rat DRG cells.
• This study provides additional molecular evidence to support the mechanisms of action of *L. rhinocerotis* as smooth muscle relaxant.
• This data is useful for future mechanistic study design aiming to elucidate the full mechanisms of action of *L. rhinocerotis*.

## 1 Data

**Fig. 1:**
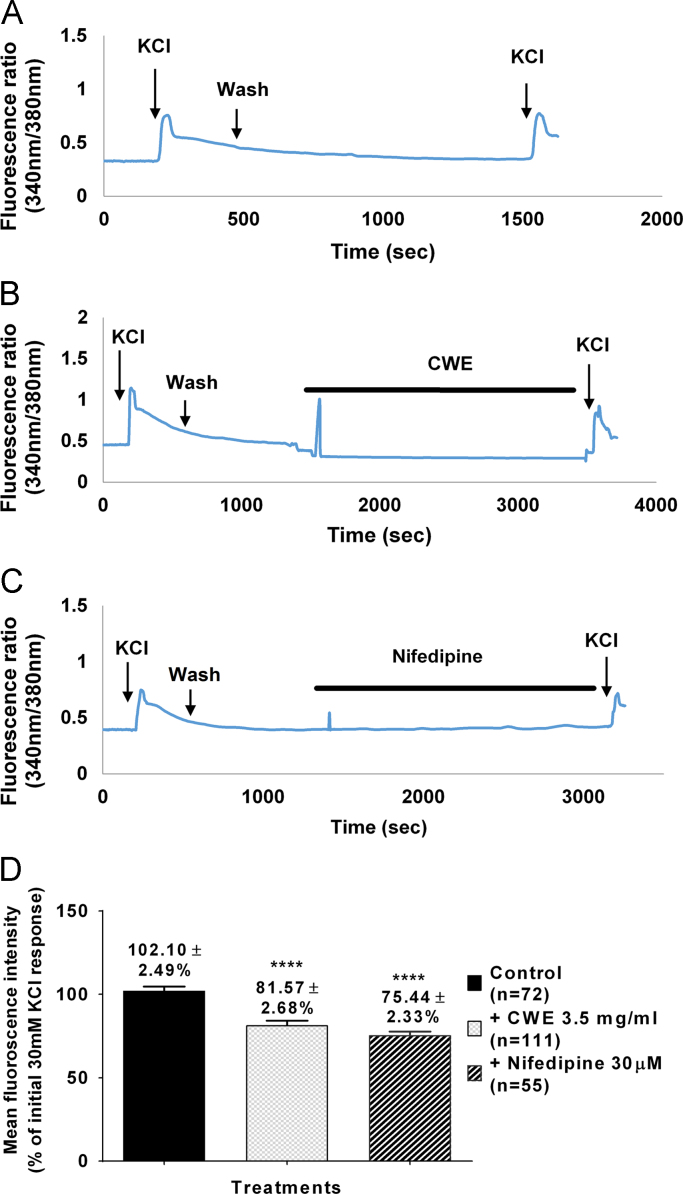
Representative calcium imaging traces of (A) control, (B) CWE (3.5 mg/ml), (C) nifedipine (30 μM), -treated rat dorsal root ganglion neuronal cells. Trace (A) shows similar magnitude of increase in Ca^2+^ transients (KCl peaks) following first and second 30 mM KCl application. The second KCl peak was attenuated following incubation with (B) 3.5 mg/ml CWE and (C) 30 μM nifedipine (incubation period shown by thick bar). Artefact spikes were observed at 1500 s in trace (B) and (C) slightly after the start of compound infusion. Figure (D) shows the effects of control (*n*=72), CWE (*n*=111) and nifedipine (*n*=55) on the second KCl peak. The data represents the mean values±SEM of n number of neurons. One-way ANOVA where comparison of mean was made between control and treatment groups, ****p<0.0001.

The intracellular Ca^2+^ [(Ca^2+^)_i_] level of DRG neuronal cells were calculated as the change in the ratios of peak fluorescence intensities (measured at 500 nm) at excitation wavelengths of 340 and 380 nm respectively. Addition of KCl serves to depolarize the DRG neuronal cells. The first addition of 30 mM KCl (represented as the initial KCl peak response) evoked an initial (Ca^2+^)_i_ increase. This was followed by a washed out period. Changes in (Ca^2+^)_i_ were expressed as the percentage of the initial KCl response. The second KCl peak response in the control group remained similar as the first KCl peak (102.10±2.49%, *n*=72; Fig. 1A and D). Pre-incubation of cells with CWE for 30 min, attenuated the increase of (Ca^2+^)_i_ evoked by KCl exposure (81.57±2.68, *n*=111; Fig. 1B and D). Similar reduction was observed with nifedipine (75.44±2.33, *n*=55; Fig. 1C and D).

## 2 Experimental design, materials, and methods

### 2.1 *L. rhinocerotis* CWE extraction

Cultivated ground sclerotial powder of *L. rhinocerotis* (TM02 cultivar) was supplied by Ligno Biotech Sdn. Bhd. (Selangor, Malaysia). The preparation method *L. rhinocerotis* CWE is reported in our previous paper [1]. The percentage yield of the CWE was 10% (w/w).

### 2.2 Coverslip preparation and coating

Glass coverslips were sterilized in the following steps: Coverslips were washed in 10% Decon solution for at least 2 h, rinsed for five times with double distilled water (ddH_2_O), soaked in 4 M concentrated HCl for at least 30 min, rinsed again for five times with ddH_2_O, and finally autoclaved before coating. To coat the coverslips, 100 µl of poly-L-Lysine was added to the centre of the coverslips and left for at least 40 min at room temperature. The coverslips were then washed ddH_2_O and allowed to dry. 10 µl of Laminin was added, followed by drying for at least 45 min. Coverslips were washed again with ddH_2_O and allowed to dry before use.

### 2.3 Dorsal root ganglia (DRG) cell preparation

Male Sprague Dawley rats were purchased from Charles River, UK. The initial weight range was 200–250 g. Studies were carried out in accordance with the UK Home Office Animals (Scientific Procedures) Act (1986). The use of the animals was approved by the University of Nottingham (UK) Animal Welfare and Ethical Review Body, approval reference number 000100. Rats were group housed at the Bio Support Unit, University of Nottingham, in open cages and fed ad libitum. The rats were sacrificed by concussion of brain, followed by cervical dislocation. Spinal column and the dorsal root ganglia (DRG) neurons were isolated from adult male Sprague Dawley rats following the previously described method [2]. The DRG neurons are put into Dulbecco's calcium- and magnesium-free phosphate-buffered saline solution (PBS) and washed once by gravity. Then, 3.5mls of Collagenase solution [Neurobasal media 90% (Invitrogen, UK); Horse serum 10%; Collagenase] was added to the DRG neurons and incubated for 90 min at 37 °C. The DRG neurons were washed gently in PBS for three times by gravity. 0.25% Trypsin solution was added to the DRG neurons and the neurons were gently pipetted up and down so to partly disrupt the ganglia. Subsequently, the neurons were incubated for 10 min at 37 °C. 1 ml of BSA solution (16% w/v in HBSS-HEPES) was added to the DRG neurons and triturated more firmly so to totally dissociate the DRG cells. The cell suspension was then carefully layered on top of 3mls of the BSA solution, followed by centrifugation at 1600 rpm for 6 min. The supernatant solution was carefully removed. The cell pellet was gently resuspended in 170 µl of the complete neurobasal media. 20 µl of cells were pipetted onto each coverslip and incubated at 37 °C, 5% CO_2_. After 15–20 min of incubation (once cells have attached), 130 µl of complete neurobasal media was added to the coverslip and cells were incubated overnight.

### 2.4 Fura 2-AM loading

Prior to imaging, a fluorescent calcium dye, Fura 2-AM, was loaded into the cell in order to visualize the calcium dynamics. Coverslip with cells was removed from incubator and rinsed three times with superfusion buffer. Fura 2-AM solution was immediately added to the cells and incubated for 30 min in dark. Cells were then washed three times with superfusion buffer and left for 15 min before the experiment.

### 2.5 Calcium imaging experiment

Coverslips were fixed on a Perspex chamber using vacuum grease. DRG neurons were superfused (2 ml/min) continuously and the first 30 mM KCl (represented as the initial KCl response) was added to evoke depolarization-induced Ca^2+^ influx. This was followed by a 20-min washout period with superfusion buffer. 30 mM KCl was added again to the same preparation to evoke a second KCl peak response in the control set (without any treatment). Similar experiments were repeated for the treatment sets, in which cells were pre-incubated with CWE 3.5 mg/ml or nifedipine 30 µM for 30 min, prior to second KCl addition.

### 2.6 Data analysis

Images were taken using a Leica DM-IRB and DM-IL microscope (Leica microsystems, Germany) equipped with QImaging Retiga-EXi camera. The (Ca^2+^)_i_ of individual neurons were estimated as the change in the ratios of peak fluorescence intensities (measured at 500 nm) at excitation wavelengths of 340 and 380 nm respectively. The mean 340/380 nm ratio represents the (Ca^2+^)_i_ while the changes in (Ca^2+^)_i_ were expressed as the percentage of the initial KCl response. Neurons that displayed <10% of KCl responses were treated as non-responsive to drug. The *n* number represents the number of neuron cells tested. Data were expressed in mean values±SEM. Statistical analysis was performed using one-way ANOVA. *P*<0.05 was considered statistically significant.

### 2.7 Drugs and materials

All chemicals and reagents were purchased from Sigma-Aldrich (UK) unless stated otherwise. Superfusion buffer was prepared following composition (in mM): NaCl 145; KCl 5; CaCl_2_ 2; MgSO4 1; HEPES 10; glucose 10; and BSA 0.1% at pH 7.4. Buffer was kept constantly at 37 °C during use. Fura 2-AM was dissolved in anhydrous DMSO to make a stock concentration of 5 µM. CWE was dissolved in purified water to make stock concentration of 100 mg/ml prior to the experiment. Nifedipine was dissolved in DMSO to make stock concentration of 0.1 mM (0.033% v/v in DMSO).
