# Supplementary material for: Data on the Lignosus rhinocerotis water soluble sclerotial extract affecting intracellular calcium level in rat dorsal root ganglion cells
Source: Data Brief. 2018 Apr 14;18:1322–6. doi: 10.1016/j.dib.2018.04.033 (PMC5997891; doi:10.1016/j.dib.2018.04.033)
Supplement: Supplementary file 1 — Supplementary material [file mmc1.docx]

**Title: Data on the *Lignosus rhinocerotis* water soluble sclerotial extract affecting intracellular calcium level in rat dorsal root ganglion cells**

**Conflict of interest**

Ligno Biotech Sdn Bhd supplied the sclerotial powder of Lignosus rhinocerotis. The authors declare that the research was conducted in the absence of any commercial or financial relationships that could be construed as a potential conflict of interest.
